# Development of a nomogram for predicting the high-risk groups of solid-pseudopapillary neoplasms of the pancreas

**DOI:** 10.3389/fonc.2023.1297497

**Published:** 2024-03-15

**Authors:** Xiaocheng Li, Jianji Ke, Xinlun Dai, Liang Guo, Li Zhang, Yahui Liu, Bai Ji

**Affiliations:** ^1^ Department of Hepatobiliary and Pancreatic Surgery, General Surgery Center, First Hospital of Jilin University, Changchun, China; ^2^ Department of Pathology, First Affiliated Hospital of Jilin University, Changchun, China; ^3^ Department of Radiology, First Affiliated Hospital of Jilin University, Changchun, China

**Keywords:** solid pseudopapillary neoplasms of the pancreas, malignant behavior, nomogram, PNI, clinical symptoms

## Abstract

**Background:**

Solid pseudopapillary neoplasms (SPNs) of the pancreas are indolent rare tumors with malignant potential. The risk factors associated with the malignant behavior of SPNs are still unclear.

**Methods:**

A retrospective analysis of patients with SPNs who underwent surgical treatment in the First Hospital of Jilin University from January 2010 to January 2022 was conducted. The clinical baseline data, pathology, imaging, and laboratory indicators of the patients were analyzed by univariate and multivariate logistic regression to identify the independent risk factors associated with the high-risk groups, and a predictive model was established in the form of a nomogram.

**Results:**

In multivariate analysis, clinical symptoms (P < 0.001), unclear tumor margins (P = 0.001), incomplete tumor capsules (P = 0.005), maximum tumor diameters ≥ 7.2 cm (P = 0.003), and prognostic nutritional index values < 47.45 (P = 0.007) were independent risk factor for SPNs with high-risk groups. A nomogram model was successfully established to predict high-risk groups of SPNs. The area under the receiver operating characteristic curve was 0.856. The calibration prediction curve was in good agreement with the standard curve.

**Conclusion:**

The nomogram model based on clinical symptoms, inflammatory markers, and imaging features had a high application value in the preoperative prediction of the high-risk groups of SPNs. A novel nomogram of the affiliated hospital of Jilin University-SPNs risk model was proposed for routine application to guide the patient counseling in clinical practice.

## Introduction

Solid pseudopapillary neoplasms (SPNs) of the pancreas were first described by Frantz in 1959, yet nearly 100 years later, the question of whether these tumors are benign or malignant remains unanswered (1). Selective surgical resection and monitoring are forms of intervention used to prevent malignant behavior and most patients with SPNs have a good prognosis (2, 3). SPNs are a type of pancreatic cystic tumor, and European guidelines for the treatment of cystic pancreatic tumors recommend standard resection (4). Therefore, radical resections such as pancreaticoduodenectomy and distal pancreatectomy with/without splenectomy are usually preferred during the past decade (5, 6). However, the applicability of standard resection to SPNs therapy remains unclear, as most SPNs do not have malignant behavior and these lesions may be overtreated to result in a high rate of morbidity and long-term endocrine/exocrine dysfunction due to wide resection of the pancreatic parenchyma (5, 7). With increasing knowledge about the diagnosis and differential diagnosis of patients with SPNs lesions, the popular approach has shifted from open to laparoscopic surgery, from standard pancreatic resection to minimal tissue-sparing resections (such as enucleation) (8, 9). However, given the malignant potential of these tumors, enucleation does not ensure oncological safety, and it is difficult to clinically distinguish benign from malignant SPNs (10). The optimal surgical procedure for patients must be based on safety. However, the optimal surgical procedure for SPNs are controversial. Thus, determining the appropriate extent of resection has been a challenge for surgeons (1). Predicting which groups have or are likely to have malignant behavior in the future is of interest because malignant behavior is associated with a high risk of SPNs recurrence and poor prognosis (11), and some studies suggest that more complete resection and closer follow-up should be considered (12–15). Choosing the appropriate surgical approach that will benefit patients with SPNs and which patients require close monitoring after surgery is currently challenging, as clinical decisions are often based solely on the imaging characteristics of the lesion. Imaging showing high-risk features and features of concern accordingly indicated the need for more thorough surgical intervention or follow-up (16). However, the sensitivity of these imaging features in detecting the early malignant behavior of SPNs lesions is limited and varies depending on the imaging modality analysis. Guidelines for the treatment of cystic disease of the pancreas state a requirement for the risk stratification of the malignant potential based on the presence or absence of symptoms and high-risk features on cross-sectional images. These guide clinicians to use a systematic approach to establish a diagnosis and determine individualized treatment (17), and it gave our study an idea to study SPNs in terms of clinical symptoms.

However, the largest clinical case series of SPNs currently available demonstrates that no clinicopathological factor could reliably predict clinical recurrence or metastasis after resection (10). Our scientific research centers have proven the same result. On the contrary, there are some research centers that suggest the presence of clinicopathological factors associated with the aggressiveness of SPNs (18). After further research, the results of our analysis may be attributable to the fact that the sample of recurrences and metastases was too small to obtain a positive result.

Therefore, we introduced the concept of the high-risk groups to expand the sample size of the positive group according to TNM staging. The purpose of this study was to develop and assess the value of a nomogram based on a patient’s clinical symptoms, inflammatory markers, and imaging indices in predicting the high-risk groups of a solid pseudopapillary neoplasm of the pancreas preoperatively. This information may help clinicians develop individualized treatment modalities and monitoring plans to manage patients more competently.

## Methods

### Definition of terms

In this study, the high-risk groups was defined as SPNs that either locally invaded adjacent structures, developed recurrence, or had systemic metastasis, either at the initial diagnosis or later. The low-risk groups was defined as the absence of these features at the last follow-up visit.

The inflammatory markers evaluated were the neutrophil-to-lymphocyte ratio (NLR), platelet-to-lymphocyte ratio (PLR), systemic immune-inflammation index (SI), lymphocyte-to-monocyte ratio (LMR), systemic inflammatory response index (SIRI), and prognostic nutritional index (PNI). LMR was calculated as lymphocyte count/monocyte count, NLR as neutrophil count/lymphocyte count, PLR as platelet count/lymphocyte count, SII as platelet count × neutrophil count/lymphocyte count, SIRI as neutrophils × monocytes/lymphocytes, and PNI as albumin level (g/L) + 5 × total lymphocyte count (10^9^/L).

By transforming the continuous variables into categorical variables, we significantly reduced the multicollinearity among the indicators. After the multicollinearity analysis, the vif values of all indicators were <5 (**Table 1**), so there was no serious multicollinearity among the indicators.

**Table 1:** Variance inflation factor of inflammatory index.

Variables	VIF
**SII**	2.878
**SIRI**	2.839
**NLR**	1.46
**LMR**	1.27
**PLR**	1.174
**PNI**	1.064

VIF, Variance inflation factor.

### Data collection

**Figure 1:**
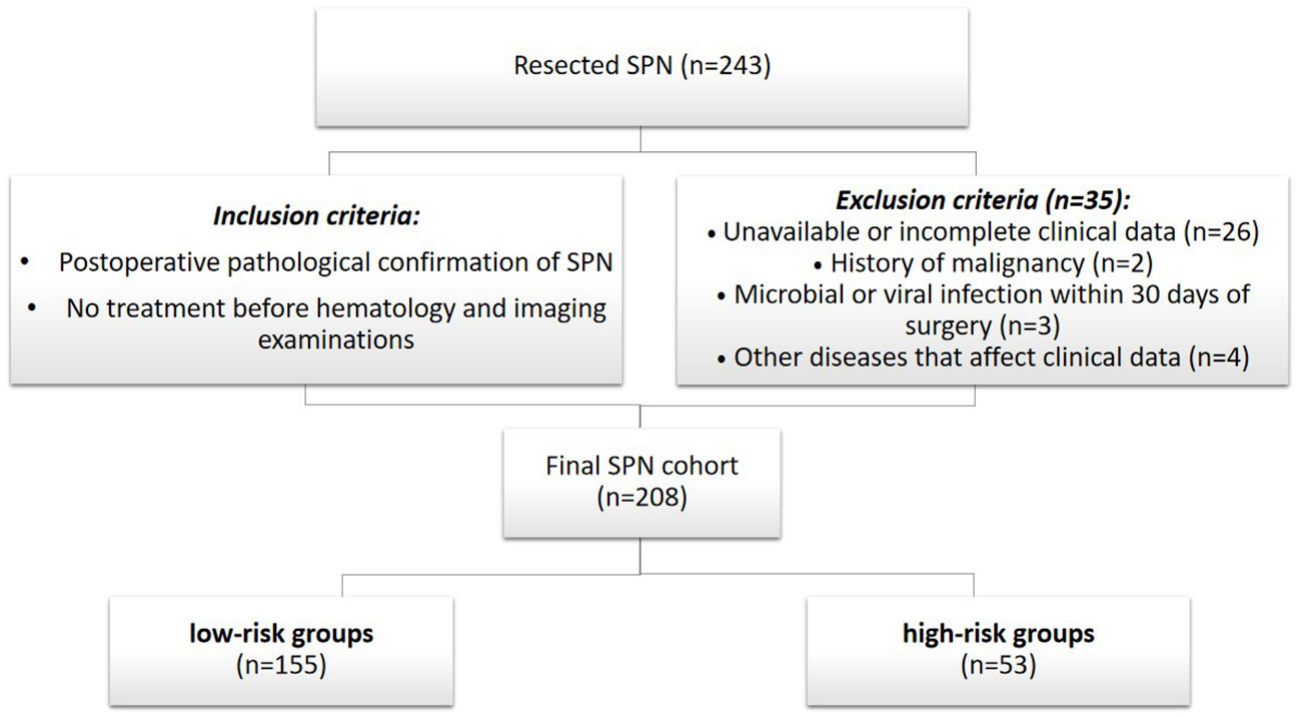
Study cohort selection flowchart.

This study followed the Declaration of Helsinki. Because of the retrospective nature of the study, patient consent for inclusion was waived. Data were selected from January 2010 to January 2022 by referring to SPNs patient records from the Jilin University First Hospital patient registry database. The records were then reviewed for a pathologically confirmed diagnosis of pancreatic SPNs. Perioperative data, including inflammatory markers, viral hepatitis, tumor markers, age, sex, symptoms, location, size, margins, calcification, pancreaticobiliary duct dilatation, distal pancreatic atrophy, capsule, date of surgery, and type of surgery were retrospectively analyzed, and coded on a spreadsheet. Strict inclusion and exclusion criteria were developed to ensure homogeneity in the preoperative inflammatory cell counts and imaging in the cohort. The inclusion criteria were (1) postoperative pathological confirmation of SPNs and (2) no treatment before hematology and imaging examinations. The exclusion criteria were (1) unavailable or incomplete clinical data (n = 26), (2) history of malignancy (n = 2), (3) microbial or viral infection within 30 days of surgery (n = 3), and (4) other diseases that affect clinical data (n = 4) (**Figure 1**). To ensure that all patients enrolled in our study accurately met the inclusion criteria, all medical records were independently reviewed by four physicians, by carefully documenting personal and family medical history, biometric parameters, and clinical information. All blood samples used for this study were collected in the morning from fasted patients as part of the routine preoperative evaluation of patients scheduled for elective pancreatic surgery. Adherence to this protocol ensured the standardization of the median blood test values. The median time between sample collection and surgery was three days (range, 1 – 6 days). All pathological findings were recorded by two physicians.

### Image analysis

All imaging scans were performed with a median time of 5 days (range, 2 – 11 days) before surgery and were recorded and analyzed by two physicians with 12 and 25 years of experience in abdominal imaging, respectively who were aware of pancreatic lesion but blinded to the clinical information, CT diagnosis, and histopathologic findings.

Qualitative analysis included the following parameters:

(a) tumor site (head-neck, or body-tail); (b) tumor size;(c) tumor margins (well defined or ill-defined); (d)presence of complete capsule; (e) presence of calcification; (f) presence of pancreatic duct dilatation;(g) pancreatic parenchyma atrophy. Any discrepancy was resolved by consensus.

### Statistical analysis

**Figure 2:**
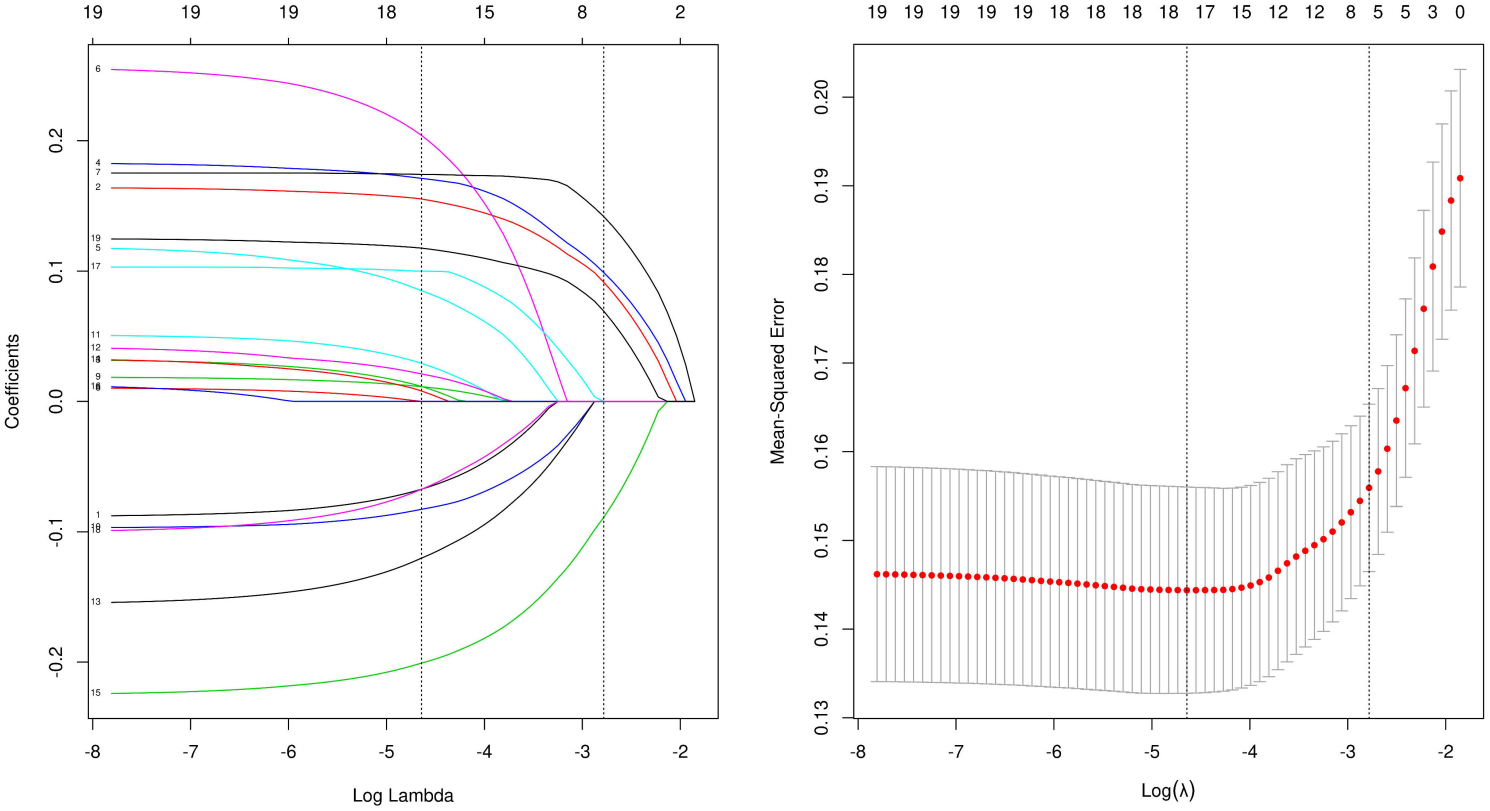
Lasso analysis.

SPSS 26.0 and R 3.6.3 software were used for the statistical analyses of the patient data. The data are described as the median and interquartile range for measured data and by number and percentage for counted data. The T-test, chi-squared test, or rank sum test was used to compare the clinical data of the patients. LASSO regression analysis was used for data dimensionality reduction and element selection (**Figure 2**). Single- and multi-factor logistic regression was used to identify the independent risk factors associated with SPNs with high-risk groups, of which OR>1 results indicated that the variable was a risk factor. Cutoffs were determined by transforming continuous information into categorical variables based on the ROC’s maximum Youden index (sensitivity plus specificity minus 1). A predictive model was developed in the form of a nomogram and the accuracy of the model was verified by 1000 bootstrapping C-index and calibration plots. All statistics were considered statistically significant at P < 0.05.

## Results

### Patient demographics

A total of 208 patients were included in the study (**Figure 1**). All patients have received an average of 88 months of follow -up (range, 10- 152 months). **Table 2** shows a summary of the basic patient information. The ratio of women to men was 177:31 (or 5.71:1), and the mean age of the participants was 31.0 years (range, 8 – 65 years). At the time of consultation, 117 (56.3%) patients presented with clinical symptoms, including abdominal pain (n = 88), bloating or abdominal discomfort (n = 19), nausea and vomiting (n = 6), and vomiting of blood (n = 4). Eleven (5.2%) patients had viral hepatitis, including hepatitis B (n = 10) and C (n = 1); 25 (11.8%) had altered tumor markers, including CA199 (n = 8), CA724 (n = 5), CA242 (n = 1), CA125 (n = 5), and neuron-specific enolase (n = 6), but none of them exceeded three times the normal value. None of the patients had jaundice or bilirubin changes. Preoperatively, 164/208 patients were correctly diagnosed with SPNs from imaging. All patients in the low-risk groups, who underwent surgery with negative tumor margins, had no tumor recurrence or disease-related deaths until the last follow-up. Patients in the high-risk groups had three recurrences (6%) and five (9.4%) disease-related deaths.

**Table 2:** The comparison of baseline characteristics of all patients by group.

Variables	Classification	low-risk groups (n= 155) n (%)	high-risk groups (n=53) n (%)	P-value
Gender	Female Male	129(83.226) 26(16.774)	48(90.566) 5(9.434)	0.195
Age (years)	<25 ≥25	50(32.258) 105(67.742)	24(45.283) 29(54.717)	0.087
Symptomatic	- +	84(54. 194) 71(45.806)	7(13.208) 46(86.792)	<0.001
The maximum diameter (cm)	<7.2 ≥7.2	109(70.323) 46(29.677)	21(39.623) 32(60.377)	<0.001
Location	Head Neck Body Tail	36(23.226) 19(12.258) 39(25. 161) 61(39.355)	10(18.868) 12(22.642) 8(15.094) 23(43.396)	0.163
Margin	Clear Not Clear	98(63.226) 57(36.774)	15(28.302) 38(71.698)	<0.001
Calcification	- +	61(39.355) 94(60.645)	16(30. 189) 37(69.811)	0.233
Capsule	Complete Incomplete	102(65.806) 53(34. 194)	15(28.302) 38(71.698)	<0.001
Distal pancreatic atrophy	No Yes	110(70.968) 45(29.032)	45(84.906) 8(15.094)	0.044
Pancreatic and bile duct dilation	No Yes	116(74.839) 39(25. 161)	36(67.925) 17(32.075)	0.327
Tumor markers	- +	139(89.677) 16(10.323)	44(83.019) 9(16.981)	0.198
Viral hepatitis	- +	149(96. 129) 6(3.871)	48(90.566) 5(9.434)	0.118
NLR	<1.54 ≥1.54	63(40.645) 92(59.355)	15(28.302) 38(71.698)	0.109
PLR	<117.7 ≥117.7	50(32.258) 105(67.742)	24(45.283) 29(54.717)	0.087
SII	<781.44 ≥781.44	129(83.226) 26(16.774)	38(71.698) 15(28.302)	0.069
PNI	<47.45 ≥47.45	24(15.484) 131(84.516)	22(41.509) 31(58.491)	<0.001
LMR	<5.519 ≥5.519	86(55.484) 69(44.516)	38(71.698) 15(28.302)	0.038
SIRI	<5.23 ≥5.23	127(81.935) 28(18.065)	35(66.038) 18(33.962)	0.016
ki67, n (%)	≤3% 3-5% ≥5%	130(83.871) 12(7.742) 13(8.387)	40(75.472) 7(13.208) 6(11.321)	0.367

+, yes; −, no; CI, confidence interval; NLR, neutrophil-to-lymphocyte ratio; PLR, platelet-to-lymphocyte ratio; SII, systemic immune-inflammation index; LMR, lymphocyte-to-monocyte ratio; SIRI, systemic inflammatory response index; PNI, prognostic nutritional index.

### Univariate analysis of SPNs-related malignant behavior

All variables included in the univariate analysis are shown in **Table 3**. Overall, 53 cases (25.5%) of SPNs-related high-risk groups were diagnosed by final pathology and postoperative follow-up. Among the inflammatory markers, differences in LMR, PNI, and SIRI were statistically significant between the low-risk and high-risk groups, whereas SII, PLR, and NLR were not statistically significant. Among the hematological indicators, elevated tumor markers and the presence of concomitant viral hepatitis did not differ statistically between the low-risk and high-risk groups. In preoperative imaging, large tumor size, incomplete capsules, and indistinct margins were significantly different between the low-risk and high-risk groups. In contrast, distal pancreatic atrophy and dilated pancreaticobiliary ducts were not statistically different between low-risk and high-risk groups. None of the 208 patients showed concomitant bilirubin elevations, and bilirubin levels could not distinguish between the low-risk and high-risk groups.

**Table 3:** General characteristics of the patients and univariate logistic regression analyses for screening predictors.

Variables	Classification	N	OR	95%CI	P-value
Gender	Female	177			
	Male	31	0.517	[0.188, 1.423]	0.202
Age(years)	<25	74			
	≥25	134	0.575	[0.304, 1.088]	0.089
Symptomatic	–	91			
	+	117	7.775	[3.304, 18.293]	<0.001
The maximum diameter (cm)	<7.2	130			
	≥7.2	78	3.611	[1.886,6.912]	<0.001
Location	Head	46			
	Neck	31	2.274	[0.831,6.221]	0.11
	Body	47	0.738	[0.263,2.077]	0.566
	Tail	84	1.357	[0.581,3. 173]	0.481
SIRI	<5.23	162			
	≥5.23	46	2.333	[1.158,4.699]	0.018
LMR	<5.519	124			
	≥5.519	84	0.492	[0.250,0.968]	0.04
PNI	<47.45	46			
	≥47.45	162	0.258	[0.128,0.519]	<0.001
SII	<781.44	167			
	≥781.44	41	1.959	[0.943,4.069]	0.072
PLR	<117.7	74			
	≥117.7	134	0.575	[0.304, 1.088]	0.089
NLR	<1.54	78			
	≥1.54	130	1.735	[0.880,3.418]	0.111
Pancreatic and bile duct dilation	No	152			
	Yes	56	1.405	[0.711,2.776]	0.328
Distal pancreatic atrophy	No	155			
	Yes	53	0.435	[0.190,0.995]	0.049
Viral hepatitis	–	197			
	+	11	2.587	[0.756,8.855]	0.13
Tumor markers	–	183			
	+	25	1.777	[0.734,4.302]	0.203
Capsule	Complete	117			
	Incomplete	91	4.875	[2.461,9.658]	<0.001
Calcification	–	77			
	+	131	1.501	[0.769,2.930]	0.234
Margin	Clear	113			
	Not Clear	95	4.356	[2.204,8.606]	<0.001

+, yes; −, no; CI, confidence interval; NLR, neutrophil-to-lymphocyte ratio; PLR, platelet-to-lymphocyte ratio; SII, systemic immune-inflammation index; LMR, lymphocyte-to-monocyte ratio; SIRI, systemic inflammatory response index; PNI, prognostic nutritional index.

### Multivariate analysis and predictive nomogram

**Figure 3:**
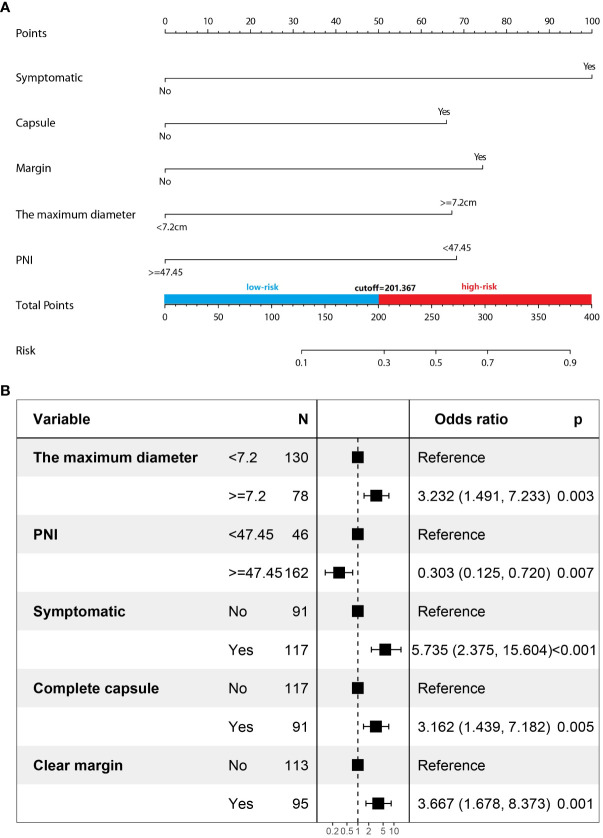
**(A)** Prediction of the high-risk groups of SPNs using a nomogram. **(B)** Model’s forest plot.

**Figure 4:**
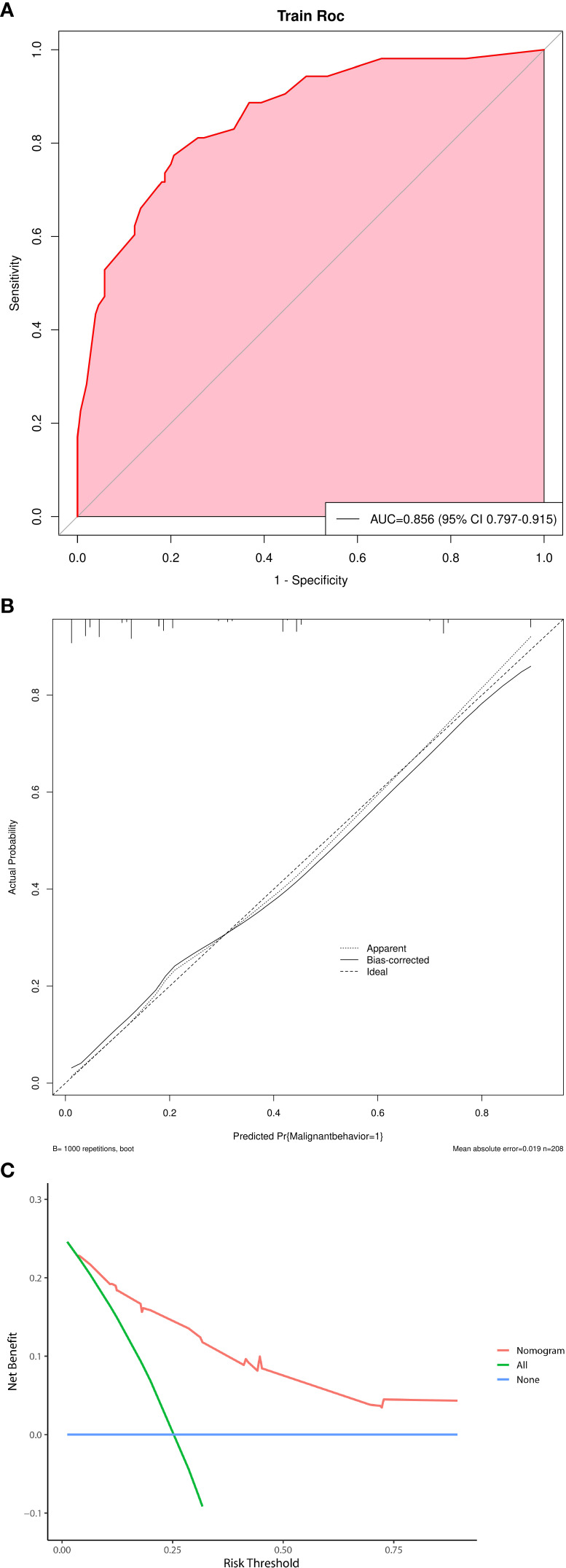
**(A)** Receiver operating characteristic curve of nomogram. AUC=0.856. **(B)** Calibration plot of nomogram. **(C)** A decision curve analysis on the nomogram of the model.

Multivariate logistic regression analysis showed that five factors were independent predictors of SPNs-related high-risk groups: clinical symptoms (p<0.001, odds ratio [OR] = 5.735, 95%confidence interval [CI]: 2.375 – 15.604), unclear tumor margins (P = 0.001, OR = 3.667, 95%CI: 1.678 – 8.373), incomplete tumor capsules (P = 0.005, OR =3.162, 95%CI: 1.439 – 7. 182), maximum tumor diameter ≥ 7.2 cm (P = 0.003, OR = 3.232, 95%CI: 1.491 – 7.233), and PNI values < 47.45 (P = 0.007, OR = 0.303, 95%CI: 0.125 – 0.720) (**Table 4**). A logistic regression model was constructed based on the above five factors, and these five factors from the logistic regression model were integrated into a nomogram (**Figure 3A**) and a model’s forest plot (**Figure 3B**). For each patient, a higher total score indicated a higher risk of SPNs-related malignant behavior. The AUC (**Figure 4A**) was 0.856 (95% CI, 0.797–0.915) and the calibration plot (**Figure 4B**) shows good calibration. A decision curve analysis on the nomogram of the model (**Figure 4C**) and the nomogram have a good predictive performance.

**Table 4:** General characteristics of the patients and multivariate logistic regression analyses for screening predictors.

Variables	OR Value	95%CI		P-value
		Lower	Upper	
Symptomatic	5.735	2.375	15.604	<0.001
The maximum diameter	3.232	1.491	7.233	0.003
PNI	0.303	0.125	0.720	0.007
Capsule	3.162	1.439	7.182	0.005
Margin	3.667	1.678	8.373	0.001

CI, confidence interval; PNI, prognostic nutritional index.

## Discussion

Solid pseudopapillary neoplasms of the pancreas are low-grade malignant tumors (2). They are usually large, well-defined, mixed solid and cystic tumors, accounting for only 1 – 2% of all pancreatic tumors and occurring mostly in young women (mean age 30 years) (17). These lesions may locally invade, metastasize, or recur in 8% to 20% of the patients (2, 17). Describing the natural course of SPNs has been difficult due to its rarity and the inconsistent correlation between pathological features and the clinical course. Complete resection was associated with lower recurrence rates, and it is often the presence of malignant behavior that leads to death (14). There were no disease-related deaths in the low-risk groups and five (9.4%) in the high-risk groups in our study. Predicting the high-risk groups of SPNs is an important goal in assessing SPNs treatment and prognosis as malignant potential has been shown to be one of the important factors affecting survival (15, 19). Based on the previous study, our study further expanded the sample size of the positive group and introduced the concept of high-risk group. This study aimed to identify perioperative factors, and stratify malignant potential based on the presence of symptoms, inflammatory markers, and tumor characteristics that could predict patients who have or will develop SPNs-related high-risk groups. The developed nomogram can help surgeons to develop individualized clinical management and monitoring strategies.

Age, gender, the presence of altered tumor markers and viral hepatitis, and the amount of postoperative ki67 protein in the patients were not significant factors identifying the high-risk groups in this study. The size of the tumor has clinical significance when identifying the high-risk groups. According to the maximum Youden index (sensitivity plus specificity minus 1), the cutoff point in this study was 7.2 cm, and the larger the tumor, the higher the malignant potential. The cutoff point of the tumor has been mentioned as 5 cm in related studies (9, 11), which may be influenced by the population of the tumor study. The presence of clinical symptoms in patients was a statistically significant factor in distinguishing between low-risk and high-risk groups. In a multifactorial analysis assessing the relationship between clinical features and malignancy in cystic disease of the pancreas, the presence of symptoms was an independent risk factor for malignancy (17). Similarly, in SPNs, symptomatic presentation was a statistically significant factor in distinguishing benign and malignant behavior of tumors in several studies (13), consistent with the results of the present study. Some studies also pointed to the need for the risk stratification of malignant potential in the management of cystic disease of the pancreas based on the presence or absence of symptoms and high-risk features on cross-sectional images to guide clinicians to have a systematic approach to establishing a diagnosis and determining individualized treatment (17). The present study also followed this suggestion and used the presence of symptoms and imaging features to plot a nomogram to predict SPNs-related malignant behavior. In a related study, neither age (20), gender (13, 21), nor amount of ki67 (13) in the patients could distinguish between benign and malignant tumors, consistent with the results of the present study. Tumor-associated inflammation and nutritional status have a significant impact not only on the occurrence of various types of pancreatic tumors (22), but also on tumor progression, the assessment of the malignancy of various types of pancreatic tumors, and the prediction of recurrence and patient prognosis (23, 24).

Several inflammatory and nutritional biomarkers, such as NLR, PLR, SII, and PNI are widely used to assess patient conditions and prognosis. These parameters can easily be implemented in a routine check. However, there are fewer studies on the relationship between SPNs and inflammatory and nutritional indicators. The relationship between inflammatory markers and SPNs has also been reported. Yang et al. highlighted the supportive role of preoperative NLR in predicting malignancy and recurrence‐free survival in patients with SPNs (25). Song et al. concluded that preoperative PNI was a reliable indicator of the aggressive natural history of SPNs and that patients with a high PNI and an intact envelope had the best prognosis, whereas those with low PNI values and incomplete capsules had the worst prognosis (22). In our study pair, inflammation markers had a good predictive value. A PNI value of < 47.45 (P = 0.007) was statistically significant in the multifactorial analysis. Decreases in the PNI may be attributable to hypoalbuminemia and/or lymphocytopenia. Patients with pancreatic tumors may present with protein malnutrition and decreased albumin (26). However, PNI decreases may also be attributed to an immune imbalance, resulting in insufficient lymphocyte-mediated immune responses against malignancies and the promotion of tumor cell progression (22). Compared to preoperative imaging studies, PNI is more readily available, convenient, and less invasive in predicting the offensive malignant behavior of this enigmatic entity. In this study, the preoperative hematological indicator PNI and radiological parameters were linked to assess SPNs-related high-risk groups in the form of columnar line graphs to guide clinical assessments. However, since the blood samples were obtained within one week before surgery, no firm conclusions can be drawn about the timing of the immune response to malignancy and whether it was the cause of tumor development or the outcome. Our institution is conducting a prospective study of the hematological indicators in all patients with cystic lesions of the pancreas who are followed in a multidisciplinary synergic approach. Linking these inflammatory markers to known radiological parameters can provide information to determine whether the onset of the immune response to SPNs occurs before or after the development of malignancy. Early preoperative nutritional intervention, including oral or intravenous Nutrition, is necessary for patients with low PNI values. However, many factors affect inflammatory markers, and the results must be interpreted with caution.

Although SPNs is a low-grade malignant tumor, it can also exhibit malignant features, including an incomplete capsule, vascular infiltration, nerve invasion, and distant metastasis (27). In the present study, 71.7% (38/53) of the tumors in the high-risk groups and 34.2% (53/155) in the low-risk groups showed incomplete or no capsule, and the difference between the two groups was statistically significant. Unclear tumor borders were reported to have a statistically significant effect on the aggressive behavior of tumors (16). In this study, 71.7% (38/53) of the tumors in the high-risk groups and 36.8% (57/155) in the low-risk groups showed unclear borders, and the difference between the two groups was statistically significant. In addition, the present study showed a statistical difference between the high-risk and low-risk groups in terms of the maximum diameter of the tumor, consistent with related studies (14, 15). The present study found no statistically significant difference between the high-risk groups and the low-risk groups in terms of tumor location, calcification, pancreaticobiliary duct dilatation, and distal pancreatic atrophy, similar to the results of related studies (16).

In this study, multifactorial logistic regression analysis showed that the presence of clinical symptoms, indistinct tumor margins, incomplete tumor capsules, a maximum tumor diameter ≥ 7.2 cm, and PNI values <47.45 were independent predictors of SPNs-related high-risk groups. Multiple nomogram models have been successfully applied to diagnose pancreatic lesions (16, 28). A nomogram can graphically present the logistic regression equation and is easy to operate and has many clinical applications. Clinicians can obtain the probability of SPNs-related high-risk groups personalized treatment and follow-up testing of patients. The area under the curve of the model was 0.856, and the model had good predictive efficiency. Despite the high predictive yield model obtained in our study, we cannot deny that predicting the aggressiveness of SPNs is still a difficult and daunting subject. Mainly attributed to the heterogeneity of SPNS, so there is hardly any study to clearly define and obtain a “gold standard”.

Our study had several limitations. Because it was a single-center retrospective study, variables may have been omitted or selection bias might have been present, which may have affected the results. Moreover, we were unable to assess continuous variables, such as increasing trends in tumor size and changes in symptoms. The patient cohort in this study was highly selected, and inflammatory markers may be influenced by a wide range of systemic diseases. Our main focus was to exclude false-positive values that would significantly bias our results. For example, inflammatory markers may be altered in patients with synchronous malignant diseases or viral infections, and thus, we applied the exclusion criteria, which also highlights the limitations of such inflammatory markers. Finally, only internal validation methods were used to verify the accuracy of the model, and a multicenter study will be performed in future work to build an external validation set to further verify the accuracy of the model.

The combination of clinical data and evolving molecular markers may also hold promise for improving the treatment of SPNs. The surgical approach is also more minimally invasive and refined. However, regardless of the type of procedure, resection should be performed to keep the patient tumor-free because negative cut margins has an extremely low rate of tumor recurrence and excellent long-term survival. Therefore, this study is meaningful to guide clinicians to develop individualized treatment modalities and monitoring plans by preoperatively predicting which patients with solid pancreatic pseudopapillary tumors have or may have malignant behavior in the future. It is expected that conservative observational treatment will be selected in the future under the condition of ensuring patient safety. A lack of understanding of the natural history of SPNs remains an additional barrier to optimal patient care. Integrative therapists, radiologists, pathologists, and surgeons are the best contributors to achieving this goal.

## Conclusion

Our study confirmed that preoperative patient-based clinical symptoms, tumor size, tumor capsule, tumor margin, and PNI values would be simple and effective tools to assess the natural history of SPNs and predict the high-risk groups. A novel nomogram of the affiliated hospital of Jilin University-SPNs risk model was proposed for routine application to guide the patient counseling in clinical practice.

## Data availability statement

The original contributions presented in the study are included in the article/supplementary material. Further inquiries can be directed to the corresponding authors.

## Ethics statement

The human studies were reviewed and approved by the Research Ethics Committee of the First Affiliated Hospital of Jilin University. This study was done in line with the Helsinki Declaration and with the informed consent of all patients.

## Author contributions

XL: Conceptualization, Formal analysis, Methodology, Writing – original draft, Writing – review & editing, Data curation, Resources. JK: Data curation, Methodology, Writing – review & editing. XD: Data curation, Software, Writing – review & editing. LG: Data curation, Supervision, Writing – original draft. LZ: Data curation, Investigation, Writing – original draft. YL: Writing – original draft. BJ: Conceptualization, Data curation, Formal analysis, Methodology, Project administration, Supervision, Writing – original draft.
